# Supplementary material for: Which factors should be included in triage? An online survey of the attitudes of the UK general public to pandemic triage dilemmas
Source: BMJ Open. 2020 Dec 8;10(12):e045593. doi: 10.1136/bmjopen-2020-045593 (PMC7725087; doi:10.1136/bmjopen-2020-045593)
Supplement: Supplementary data [file bmjopen-2020-045593supp001.pdf]

Online Supplemental Material

Supplemental Table 1.
Demographic characteristics of respondents, N – 763

Age Mean ± SD (range) 44 ± 15 (18-86)

**Sex**		
Male	43.5	332
Female	52.0	397
Other	0.3	2
Did not answer	4.2	32
**Highest level of education**		
Some college but no degree	24.9%	190
Professional degree (JD, MD)	3.5%	27
Master's degree	11.1%	85
Less than high school degree	5.4%	41
High school graduate (high school diploma or equivalent including GED)	16.6%	127
Doctoral degree	1.8%	14
Bachelor's degree in college (4-year)	27.3%	208
Associate degree in college (2-year)	9.2%	70
Did not answer	.1%	1

**Household income**		
Less than £10,000	4.9%	37
£10,000 to £19,999	15.4%	116
£20,000 to £29,999	22.1%	166
£30,000 to £39,999	15.9%	120
£40,000 to £49,999	11.8%	89
£50,000 to £59,999	8.3%	63
£60,000 to £69,999	6.5%	49
£70,000 to £79,999	4.1%	31
£80,000 to £89,999	2.0%	15
£90,000 to £99,999	1.7%	13
£100,000 to £149,999	4.3%	33
£150,000 or more	2.5%	19
Did not answer	1.6%	12

**Employment status**		
Working (paid employee)	71.0%	537
Working (self-employed)	8.6%	65
Not working (temporary layoff from a job)	3.4%	26
Not working (looking for work)	2.7%	21
Not working (retired)	7.5%	57
Not working (disabled)	1.1%	9

Not working (other)	4.8%	37
Prefer not to answer	0.5%	4
Did not answer	.9%	7

Supplemental Figure 1a

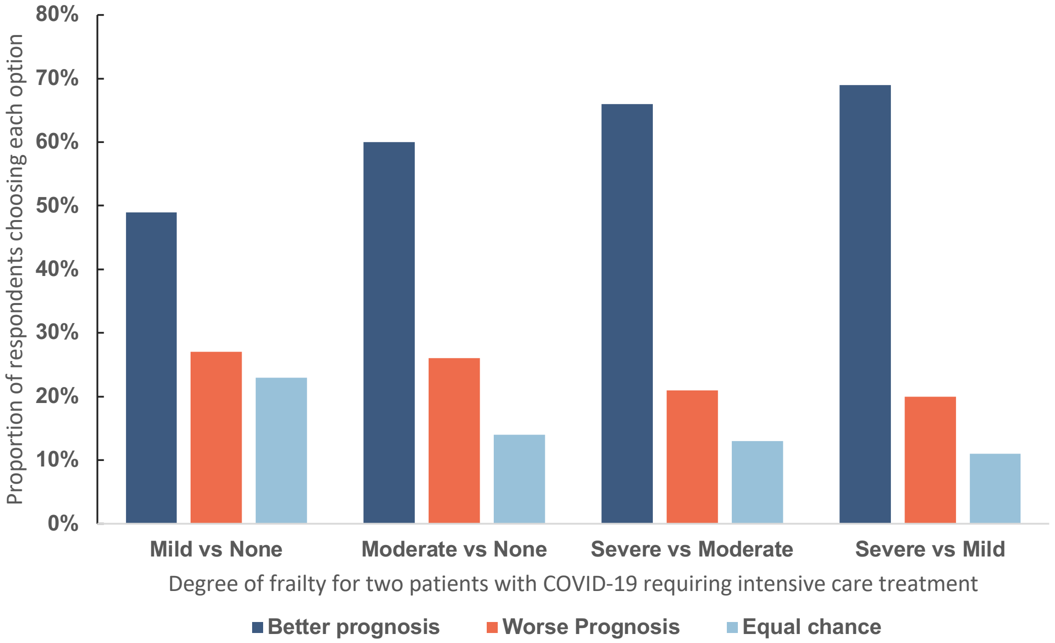

Supplemental Figure 1a*. Respondent choices in a triage dilemma involving withholding treatment from one of two patients with different degrees of pre-existing frailty but equivalent survival chances and life expectancy*.

There was a significant difference in the distribution of answers between scenarios. **1.** Severe/moderate vs severe/mild, X^2 (3, N = 763) = 6.583, p = .32 **2.** Severe/moderate vs moderate/none X^2 (3, N = 763) = 19.85, p < .001 **3.** Severe/moderate vs mild/none X^2 (3, N = 763) = 95.669, p < .001 **4.** Moderate/none vs mild/none X^2 (3, N = 763) = 69.210, p < .001.

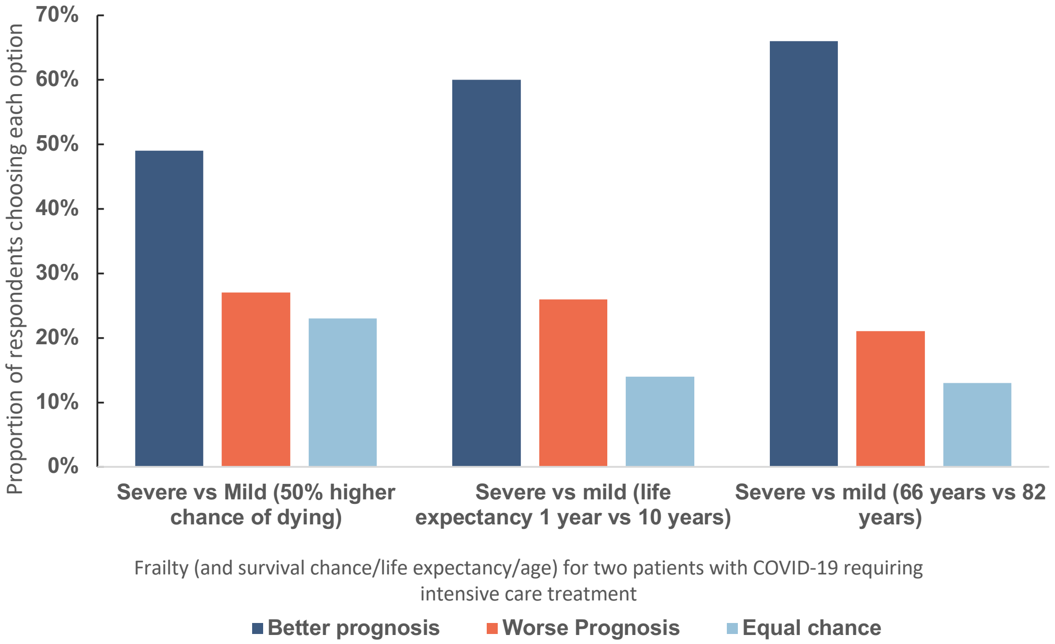

***Supplemental Figure 1b**. Respondent choices in a triage dilemma involving withholding treatment from one of two patients with different degrees of pre-existing frailty but equivalent survival chances, life expectancy or age. In the first two scenarios, the more frail patient had lower survival chance/life expectancy. In the third scenario, the more frail patient was younger.*

Severe/mild with reduced life expectancy vs same life expectancy: X^2 (3, N = 763) = 95.258, p < .001; Severe/mild with increased chance of dying vs same chance: X^2 (3, N = 763) = 20. 85, p < .001
